# Quality control of phenotypic forms data in the Type 1 Diabetes Genetics Consortium

**DOI:** 10.1177/1740774510373495

**Published:** 2010-08

**Authors:** Letitia H Perdue, Lotte Albret, Alan Aldrich, Amanda Loth, Elizabeth G Sides, Angela Dove, Ana M Wägner, Rebecca Waterman, June J Pierce, Beena Akolkar, Michael W Steffes, Joan E Hilner, the T1DGC

**Affiliations:** ^a^Division of Public Health Sciences, Wake Forest University Health Sciences, Winston-Salem, NC, USA, ^b^Hagedorn Research Institute, Gentofte, Denmark, ^c^University of Alaska Anchorage College of Arts and Sciences, Integrated Sciences, Anchorage, AK, USA, ^d^Burnet Clinical Research Unit, Walter and Eliza Hall Institute of Medical Research, Melbourne, Victoria, Australia, ^e^Benaroya Research Institute, Virginia Mason, Seattle, WA, USA, ^f^Department of Endocrinology, Hospital Universitario Insular de Gran Canaria, Las Palmas de Gran Canaria, Spain, ^g^Department of Medical and Surgical Science, Universidad de Las Palmas de Gran Canaria, Las Palmas de Gran Canaria, Spain, ^h^Division of Diabetes, Endocrinology and Metabolic Diseases, National Institute of Diabetes and Digestive and Kidney Diseases, National Institutes of Health, Bethesda, MD, USA, ^i^Department of Laboratory Medicine and Pathology, University of Minnesota Medical School, Minneapolis, MN, USA, ^j^Department of Biostatistics, School of Public Health, University of Alabama at Birmingham, Birmingham, AL, USA

## Abstract

***Background*** When collecting phenotypic data in clinics across the globe, the Type 1 Diabetes Genetics Consortium (T1DGC) used several techniques that ensured consistency, completeness, and accuracy of the data.

***Purpose*** The aim of this article is to describe the procedures used for collection, entry, processing, and management of the phenotypic data in this international study.

***Methods*** The T1DGC ensured the collection of high quality data using the following procedures throughout the entire study period. The T1DGC used centralized and localized training, required a pilot study, certified all data entry personnel, created standardized data collection forms, reviewed a sample of form sets quarterly throughout the duration of the study, and used a data entry system that provided immediate feedback to those entering the data.

***Results*** Due to the intensive procedures in developing the forms, the study was able to uphold consistency among all clinics and minimal changes were required after implementation of the forms. The train-the-trainer model was efficient and only a small number of clinics had to repeat a pilot study. The study was able to maintain a low percentage of missing data (<0.001%) and low duplicate data entry error rate (0.10%).

***Conclusions*** It is critical to provide immediate follow-up in order to reinforce training and ensure the quality of the data collected and entered.

## Introduction

The Type 1 Diabetes Genetics Consortium (T1DGC) recruited affected sibling pair (ASP) families, trio families, cases, and controls in over 200 clinics spanning four networks (Asia-Pacific, European, North American, and United Kingdom) worldwide [1]. A multi-level approach ensured that the quality of the data remained high throughout the six years of recruitment. Due to the international scope of T1DGC and the number of participating clinics, four Regional Network Centers were established (Melbourne, Australia; Copenhagen, Denmark; Seattle, Washington, USA; and Cambridge, United Kingdom). Personnel at the Regional Network Centers provided training for the clinic staff within their own networks, performed site visits as needed, reviewed completed forms, and entered study data into a central database maintained at the Coordinating Center. The Coordinating Center (Winston-Salem, North Carolina, USA) was responsible for overseeing all of the Regional Network Centers. This model, similar to that in the Evaluation of Subcutaneous Proleukin® in a Randomized International Trial (ESPRIT), allowed for consistency across all clinics and reduced the costs required for training [2]. As has become the practice in multi-center studies, the T1DGC primarily used the study website (https://t1dgc.org) to allow consistent distribution of the study forms and written procedure manuals to the Consortium Members and data collection sites [2–4].

Extensive form review at every level (clinics, laboratories, Network Centers, and Coordinating Center), feedback through a query system, and the use of dynamic reports allowed the study to identify and address data collection problems in a timely manner. Using methods such as those used in the T1DGC (*i.e.,* providing immediate feedback upon completion of data entry, requiring certification for data entry personnel, and performing duplicate data entry on a percentage of all forms), other multi-center studies have been able to maintain a low data entry error rate [5–9].

## Methods

To ensure that the data were collected consistently among the 214 clinics around the globe, standard data collection forms were used, rigorous form review was completed in multiple locations, and a data entry system was used to provide immediate feedback regarding form completion and data entry errors. Due to these intensive front-end quality control mechanisms, the phenotypic data collected by the T1DGC are exceptionally clean and complete. A description of the form sets used in the T1DGC is provided in Table 1.

### Development and distribution of forms

To standardize T1DGC data collection, the same data collection forms were used in every clinic throughout the study. Data forms were created at the Coordinating Center with input and approval of various study committees that were comprised of representatives from each of the networks (*e.g.,* the Phenotyping/Recruitment Committee, the Network Coordinators Committee, and the Steering Committee). Data forms were pilot tested by the Network Coordinators at several points during the development phase.

**Table 1:** Summary of T1DGC data collection forms

Form name	Maximum number of fields per form
*Affected sibling pair form set: Required for core family completion^a^*	
Eligibility form (completed by proband) OR eligibility form (completed by guardian)	50 or 54
Consent summary form	81
Layered informed consent (completed for proband AND affected sibling)	6
Exam form (proband)	84
Exam form (affected sibling)	46
Blood collection form (completed for proband AND affected sibling)	31
	
*Additional affected sibling pair forms^b^*	
Layered informed consent (completed for all additional family members)	6
Exam form (parent(s))	58
Exam form (unaffected sibling(s))	46
Exam form (additional affected sibling(s))	46
Blood collection form(s) (completed for all additional family members)	31
Application(s) for additional affected sibling	31
	
*Trio family form set: Required for core family completion^c^*	
Pre-eligibility form^d^	11
Eligibility form (completed by proband) OR eligibility form (completed by guardian)	30 or 33
Consent summary form	30
Layered informed consent (completed for all family members)	6
Exam form (proband)	61
Exam form (parent) (completed about father AND mother)	58
Blood collection form (completed for all family members)	31
	
*Case participant form set: Required for participant completion*	
Eligibility form (completed by case) OR eligibility form (completed by guardian)	32 or 34
Consent record	15
Exam form (case)	83
Blood collection form	31
	
*Control participant form set: Required for participant completion*	
Eligibility form (completed by control) OR eligibility form (completed by guardian)	35 or 38
Consent record	15
Blood collection form	31

a Inclusion of the proband and one affected sibling is required for the core affected sibling pair family to be considered complete.

b Inclusion of both biological parents, up to two unaffected siblings and an additional three affected siblings can be included in the  T1DGC affected sibling pair family.

c Inclusion of the proband and both biological parents are required for the trio family to be considered complete.

d The Pre-Eligibility Form is required in the North American Network. This form is not completed in any of the other networks.

The informed consent was the only document translated into the participant’s native language [10]. Each clinic was required to have at least one staff member who was fluent in both English and the native language. All other forms were in English, and clinic staff members were instructed to read the questions to the participant in the participant’s native language.

The number and length of the forms were kept to a minimum to decrease participant burden. A total of 17 forms were created for the typical family data collection: eight forms exclusively for ASP families; six forms for trio families; a layered consent form [10]; and two blood collection forms (original and re-collection). Six forms were created for the case and control data collection: two exclusively for cases; one exclusively for controls; one consent record form used for both cases and controls; and two blood collection forms, similar to those used for the family collection. Form length ranged from 2 to 10 pages and included up to 18 questions per form. Due to skip patterns, the number of data fields on each form varied. The number of data fields per form ranged from 6 to 84.

For each T1DGC family, one consent summary form and one eligibility form was completed. The consent summary form allowed the clinic staff to track the type and date of consent for all family members. One eligibility form was completed per family and it assessed the eligibility of all key family members. For each participant, an exam form and a blood collection form were completed. To decrease the amount of time the participant needed to be present in the clinic, some forms could be completed from existing records or through a telephone interview prior to the clinic visit.

As the T1DGC is primarily a family-based study, questions were phrased so that they could be asked of either the participant or the parents (if young children were participating). Questions were written as if addressing the participant, with a variation of each question in parentheses and italics, for use when questions were asked of the parent or guardian.

To emphasize responses that would make a participant or family ineligible, check boxes were shaded gray. While the use of skip patterns was minimized, their use in the data forms could not be eliminated. For example, the recruitment of parents in ASP families was encouraged, but not required. When the parents were participating, the family history was captured on the parental exam form and these questions were skipped on the proband exam form. When the parents were not participating, the proband answered the questions regarding his/her parent’s family history.

Answers to questions that would elicit different responses due to international differences in recording certain data (*e.g.,* dates and body weight) were anticipated. All dates were recorded consistently as day, month, and year, with the name of the month written out in its entirety. For questions about weight and height, clinic staff checked whether the answer was reported in pounds or kilograms, and in inches or centimeters, respectively.

‘Not applicable’ was added as an acceptable response for some questions, based on network-specific requirements. For example, ‘not applicable’ was recorded for date of birth in the United Kingdom Network as these data cannot be collected due to regulations in the United Kingdom. All networks, with the exception of the North American Network, selected ‘not applicable’ to the question regarding whether the participant was of Latino, Hispanic, or Spanish origin.

To provide easy access for all clinics across all networks, for cost-efficiency, and for ease in future changes to the forms, all forms were made available through the T1DGC website to be printed as needed by the clinic staff. Question-by-question instructions for each form were included in the manual of operations, which was also available on the T1DGC website. The exception to clinic access to the website was in the United Kingdom Network where the Network Center printed and distributed form sets to the nurses who traveled among clinics throughout a region.

### Training and pilot study

Coordinating Center staff trained personnel at each of the Network Centers in all aspects of data collection and data entry. Each Network Center was responsible for training study personnel at all clinics within their network. All networks held at least one central training session. For clinics joining the T1DGC before or after the central training, or when additional training was needed due to staff turnover, logistic, or language issues, local training was completed by Regional Network Center staff members.

Each clinic was required to complete a pilot study successfully on a mock family (usually volunteer clinic staff) prior to initiating participant recruitment. The pilot study covered all aspects of enrolling participants, including: administering individual consent; completing a full set of forms for a family or participant; and collecting, processing, and shipping blood samples. Pilot study forms were sent to the Regional Network Center for entry into the data entry system and all aspects of the pilot study were reviewed and approved by the staff of both the Regional Network Center and Coordinating Center before certifying a clinic. Feedback was provided to each clinic, noting specific problems in form completion and/or blood collection and shipping.

### Form review

Clinic staff members were asked to review the data collection forms for completion before the participant(s) left the clinic and again before shipping to the Regional Network Center for data entry. As form sets were received, Regional Network Center staff reviewed each form prior to data entry. Any needed form corrections due to missing pages, incorrect labeling, and other obvious errors were communicated to the clinic staff through email prior to data entry.

The Coordinating Center requested family form sets from each network on a quarterly basis for forms review and duplicate data entry. For each clinic, the first two form sets were requested for review; thereafter, a 5% sample of all forms completed during each quarter were re-entered and reviewed by the Coordinating Center Project Managers. Any form completion problems discovered were sent to the Regional Network Center Coordinator for communication to and correction by the clinic staff. If the data entry error rate was above 0.5% for any form, the Network Center was required to compare the entered forms with the paper forms, correct data entry as needed, and re-save all forms of that particular type for the affected quarter. Error rates were calculated based on the total number of fields per form set for each recruitment type.

### Data entry system and rules

After clinics sent the data collection forms to one of the four Regional Network Centers, the forms were entered into the data entry system through the T1DGC data entry website. This regional data entry enabled the T1DGC to train and certify a small number of individuals who became quite proficient at data entry. This strategy also permitted tighter security of phenotypic information by restricting access to the data entry website to a small number of trained staff. All data entry staff were required to complete a data entry certification packet (consisting of two complete form sets) prior to being allowed to enter the data from T1DGC forms. Each individual had to achieve 99.5% or better accuracy on both form sets to achieve data entry certification.

A directed flow of the data entry system required that the informed consent forms be entered first, followed by data from the eligibility forms. (See Hall, et al. [10] for more details about what information was entered from the informed consent forms.) Only after entry of data from these forms could the exam forms and blood collection forms for the key participants (*i.e.,* proband and affected sibling in ASP families; all participants in trio families; and cases and controls) be entered. Following entry of data from these core forms, data from the remaining exam and blood collection forms could be entered for other participants in families. Additionally, an interactive rules system [11] provided immediate feedback to the data entry personnel when attempting to add or modify data records.

**Figure 1:**
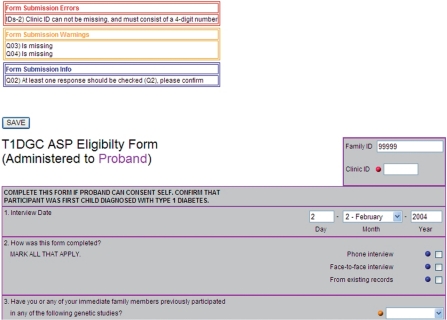
Error, warning, and informational messages in the T1DGC data entry system.

The interactive rule system allowed the data entry system to display three types of messages to the user if values were outside of a specified range or fields were left missing (Figure 1). These messages conveyed the queries that should be verified or corrected and helped to certify that the data were entered with a high degree of accuracy. Messages were displayed at the top of the data entry screen and indicated by a colored dot beside the response that was in question. ‘Informational’ messages (*e.g.,* confirm ‘Don’t Know,’ number of brothers and sisters out of range), indicated by a blue dot, were used to confirm data entered that were outside of what was normally expected for fields that were not considered key information. Informational messages did not require clinic verification, but were confirmed by the data entry staff to check that the data were entered as recorded on the form. ‘Warning’ messages, indicated by an orange dot, allowed data entry staff to save the data that they had entered, but these messages created queries on dynamic reports and in the T1DGC query system. Warning messages appeared for fields that should be corrected or confirmed by the clinic (*e.g.,* date of birth not valid, participant diagnosed outside of the expected age range). ‘Error’ messages, indicated by a red dot, displayed when key data entry fields were incorrect or missing, and the data could not be saved in the database until these errors were resolved (*i.e.,* participant ID, clinic ID, secondary ID, date of exam, or date of blood collection missing; or the indication that consent had been obtained was not checked for the key participants in the family). The number of fields that generated error messages was kept to a minimum in order to maximize the data saved at the time of initial entry.

### Dynamic reports and query system

**Figure 2:**
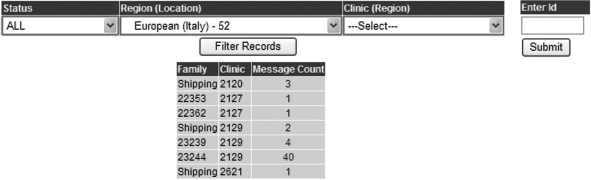
T1DGC interactive query system.

**Figure 3:**
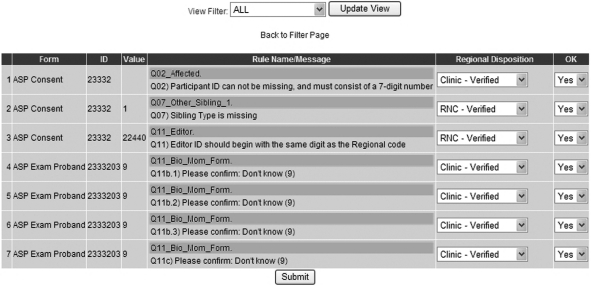
T1DGC interactive query system, examples of queries.

A final check of data entry fields was provided by using the online dynamic reports and the T1DGC query system. The dynamic reports were produced by an automated review of data that used cross-form comparisons. These reports listed samples that had not been shipped to the T1DGC laboratories, any eligibility questions, and all other irregularities. The interactive query system (Figure 2) was developed during the study to allow the Regional Network Centers to review all the network's queries or to filter the list of queries by country, clinic, or family. Queries displayed related to the messages displayed at the time of data entry as well as cross-form checks for consistency. Regional Network Center staff members were able to confirm data entered and abnormal values through the query system (Figure 3). During the development of the query system, the Network Coordinators and Coordinating Center specified the queries that could be verified at the Regional Network Center level and those that required confirmation by the clinic. Once Regional Network Center staff confirmed or corrected queries, the Coordinating Center Project Managers reviewed each response and either approved the response or requested further correction or verification at the clinic level.

Dynamic reports were created showing various levels of data and sample collection completeness for families or cases and controls. ‘Known’ families or cases and controls had some information in the T1DGC system. ‘Eligible’ families or cases and controls met the eligibility criteria, but did not have all exam information and blood samples entered into the system. ‘Completed core’ families had the information for core family members complete, but outstanding issues remained for the other family members. ‘Completed’ families or cases and controls had all forms entered and all samples received at the laboratories; however, outstanding queries remained. ‘Completed close-out’ families or cases and controls had all information in the system, all samples received at the laboratories, and no remaining queries.

When a clinic had concluded recruitment and was in the process of being closed, the Regional Network Center Coordinator and Coordinating Center Project Manager checked all reports and the T1DGC query system to verify that no remaining outstanding issues or queries remained. For a clinic to be closed, all families, cases, and controls recruited by that clinic must be identified in the dynamic reports as ‘Completed close-out.’

## Results

### Development and distribution of forms

By having the staff from the Regional Network Centers and Coordinating Center involved in the initial creation, development, and testing of the data collection forms, the forms required very few revisions so that the data were collected consistently throughout the six-year recruitment period of the project. The majority of revisions to forms after data collection began affected only the wording; only one change involved adding a variable to the database. Additional forms were created for use in special circumstances, but were not part of the standard form set.

Several of the forms required skip patterns to obtain the needed information; these patterns often created problems when the pattern was not followed at clinics and the forms had to be sent back to the clinic for correction. Additionally, one unanticipated complication that developed was when all members of a participating family did not attend the same clinic visit. When the initial participant thought his or her parents were going to participate, the family history section was skipped on the initial form. If the parents subsequently decided not to participate or could not be reached, data fields in that section were missing. To obtain this critical study information, it was decided that if the parents were not present at the initial clinic visit, the family history should be collected and recorded on the proband exam form. If the parent did participate later, the information collected from the parent (and recorded on the parental exam form) was deemed more accurate and was used in place of the information collected on the proband exam form.

### Training and pilot study

The train-the-trainer model used in the T1DGC allowed the Coordinating Center and Network Centers to modify the training to meet each network or clinic’s specific needs. When the Network Center staff performed local training, often they would remain at the clinic and oversee the completion of the pilot study. Completion of the initial pilot studies also allowed the T1DGC to determine how long it would take for samples to arrive at the network laboratories and shipping days could be modified as needed to verify that samples could be received within 24 to 48 h of blood collection. While most clinics were certified to begin data collection after a single pilot study, six (4.3%) clinics with serious errors were required to complete a second pilot study.

### Form review

As of July 4, 2009, data from a total of 50,236 forms had been entered through the T1DGC data entry system (Table 2). Due to the rigorous form review practices and the interactive ILOG JRules employed in the data entry system, the number of missing data fields was less than 0.001% (Table 3) for more than 30 million data fields.

**Table 2:** Summary of data forms entered for completed families and participants, by network and overall, T1DGC, July 4, 2009

Network	Total (*N*) ASP forms (*N* families)	Total (*N*) trio forms (*N* families)	Total (*N*) case forms (*N* cases)	Total (*N*) control forms (*N* controls)
Asia-Pacific	4725 (324)	2226 (265)	16 (16)	6 (17)
European	16,920 (1211)	115 (10)	14 (13)	6 (9)
North American	16,959 (1143)	2237 (186)	1907 (519)	2011 (692)
United Kingdom	2394 (163)	N/A	N/A	N/A
Overall	40,998 (2841)	5278 (461)	1937 (548)	2023 (718)

**Table 3:** Number of missing data fields^a^, by network and overall, T1DGC, July 4, 2009

Network	ASP forms	Trio forms	Case forms	Control forms	All forms
Asia-Pacific	15	6	0	0	21
European	13	2	0	0	15
North American	198	9	88	82	377
United Kingdom	16	N/A	N/A	N/A	16
Overall^b^	242	17	88	82	429

a The percentage of missing data fields was less than 0.001% for each form type.

b The total number of fields for each type of data form set was: affected sibling pair (ASP) = 27,519,470; trio = 2,103,645; case = 395,760; control = 147,679; and overall = 30,166,554.

The overall time from recruitment to data entry was roughly 50 days for ASP and trio family forms, although this varied by network. The overall time from recruitment to data entry was lower for the case and control forms (Table 4).

From the form review completed at the Coordinating Center, a few key issues were identified that appeared consistently across all the networks; these were communicated to the Network Coordinators so that they could work with the clinic staff. The Regional Network Coordinators also were encouraged to highlight these problems in subsequent training of personnel at new clinics to prevent their continuing occurrence. The frequency of these problems decreased in clinics where the study personnel were trained later in the study. The most common problems identified included not following skip patterns and not following instructions for making corrections to the forms.

**Table 4:** Summary statistics for time (days) from data collection to data entry, by network and overall, T1DGC, July 4, 2009

Network	ASP forms mean ± SD (median, range)	Trio forms mean ± SD (median, range)	Case forms mean ± SD (median, range)	Control forms mean ± SD (median, range)
Asia-Pacific	43.9 ± 82.8 (21,932)	43.4 ± 63.4 (27, 1181)	15.8 ± 2.2 (15.5, 6)	14.5 ± 1.6 (14.5, 3)
European	76.5 ± 125.4 (40, 1569)	170.4 ± 139.8 (161, 614)	433.1 ± 131.6 (455, 565)	456.0 ± 0.0 (456, 0)
North American	27.6 ± 55.8 (11, 1100)	50.5 ± 86.6 (15, 635)	20.5 ± 30.0 (11, 242)	19.9 ± 34.2 (11, 357)
United Kingdom	47.0 ± 59.4 (28, 696)	N/A	N/A	N/A
Overall	50.7 ± 96.4 (22, 1570)	49.6 ± 79.0 (24, 1186)	23.7 ± 48.2 (11, 721)	21.2 ± 41.6 (11, 456)

Very early in the study, the Coordinating Center discovered deviations from the study protocol at one network when reviewing the data collection forms. It was found that the data in the data entry system did not match the source document. When this discrepancy was discovered, all data for this network were marked as invalid in the data entry system and the Regional Network Center data entry staff re-entered data from all forms exactly as they had been recorded and requested corrections from the clinics when appropriate. Forms review following re-entry confirmed that the network center staff had entered the data correctly.

### Data entry system and rules

The Coordinating Center certified 22 Network Center staff members in data entry. Two staff members were required to complete a second certification due to an accuracy rate lower than 99.5%.

As noted earlier, the Coordinating Center staff entered data from a 5% sample of data collection forms from each network every quarter. The duplicate data entry system allowed for a comparison of these data to the original data entered at the Regional Network Centers; an overall and form-specific entry error rate was calculated. Whenever the data entry error rate was higher than 0.5% on any particular form, the Regional Network Center staff compared all paper forms of that type from that quarter with the study database. In only 27 instances throughout the duration of the study and across all networks was this type of review required. In the vast majority (*N* = 384), no data entry errors were found. The overall data entry error rate throughout the study was 0.1% (Table 5).

**Table 5:** Data entry error rates per data entry field (%) by quarter and overall, T1DGC, July 4, 2009

Year	1	2				3				4				5			Overall
Quarter	4	1	2	3	4	1	2	3	4	1	2	3	4	1	2	3	

Network																	
Asia-Pacific	0.61	0.11	0.05	0.03	0.06	0.00	0.05	0.33	0.07	0.15	0.03	0.14	0.03	0.00	0.00	0.09	0.10
European	0.02	0.04	0.08	0.03	0.00	0.11	0.04	0.08	0.06	0.11	0.02	0.04	0.07	0.03	0.42	0.08	0.06
North American	0.07	0.06	0.03	0.20	0.11	0.17	0.11	0.05	0.17	0.07	0.13	0.15	0.15	0.03	0.03	0.23	0.11
United Kingdom	0.14	0.31	0.82	0.11	0.02	0.08	N/A	N/A	N/A	N/A	N/A	N/A	N/A	N/A	N/A	N/A	0.16
Overall	0.19	0.11	0.11	0.09	0.05	0.11	0.07	0.11	0.11	0.10	0.09	0.11	0.12	0.03	0.18	0.13	0.10

### Dynamic reports and query system

The dynamic reports were created with the input of the Regional Network Centers; however, the T1DGC query system was introduced after several of the Regional Network Centers had procedures in place for addressing queries. Because of the delay in implementation of the T1DGC query system, there were a large number of queries that had to be resolved. In order to identify the queries that were more critical, the Coordinating Center Project Managers and Regional Network Coordinators reviewed all queries and identified the questioned items that could be verified and those that had to be corrected by the clinic personnel. This review decreased the number of queries sent to the clinics to a manageable number, without jeopardizing the quality of the data.

## Discussion

To ensure that the quality of phenotypic data was consistent and accurate across multiple clinics from countries around the world, an effective and efficient plan for data collection, data entry, and data verification had to be in place. It was critical to have input from representatives from each participating network to assure consideration of cultural factors. Due to time constraints and staggered entry of clinics into the study, it was not possible to obtain feedback from every clinic before data collection began. However, changes to the plan were made based on feedback from members of the Network Centers, Steering Committee, and Phenotyping/Recruitment Committee. Feedback from the initial clinics and the Regional Network Centers allowed the study to identify issues with forms completion and to revise the forms prior to their implementation.

Although the number of forms was kept to a minimum, clinic staff still found the completion of a form set to be a time-consuming task. The North American Network consistently had a lower time from collection to entry due to the shorter distance from the clinics to the Network Center and the use of courier services (as opposed to regular post) to ship data collection forms. Some delays were due to missing key information; forms could not be entered until this information was provided and sent to the Regional Network Centers.

Through regular site visits to the Regional Network Centers and form review, the Coordinating Center was able to monitor the quality and accuracy of the study data, ensuring that corrections were made when needed by using appropriate procedures. The low data entry error rate can be attributed to the rigorous form review and the implementation of the ILOG JRules system that flagged potential problems early during the data entry process.

The query system was not developed early enough in the data collection process to permit standardizing the procedures for handling queries across the networks. By requiring verification through the T1DGC query system after network-specific procedures had been established, the query system was viewed more as a burden than as a useful tool. To decrease the burden, the T1DGC identified specific critical queries that required verification and stopped requiring verification of noncritical fields. Implementation of procedures designed to assure the consistency, completeness, and accuracy of the data should occur early in any study and be uniform across all clinics.

The distribution of tasks between the Coordinating Center, Network Centers, and clinics allowed each T1DGC member to contribute to the collection of high quality data. Due to the standardization of data collection and extensive quality control checks, the phenotypic data collected by the T1DGC are accurate and complete. The high quality of the T1DGC phenotypic database will permit reliable interpretation of other study findings and provide the basis for meaningful publications well into the future.

## Conclusions and recommendations

Conclusions and recommendations from the T1DGC apply to many multi-center studies. Close, immediate follow-up regarding performance is important to clinics. Site visits should be conducted early in the study in order to identify and rectify any problems quickly and efficiently. It is critical to receive input from all networks in order to verify that the forms are sensitive to social and cultural influences and that the data are collected accurately.

The data cleaning and verification process should be developed in conjunction with the data collection forms. Centralized data entry allows a minimal number of people to be trained and monitored and can reduce data entry error rates.

By employing extensive checks through the data entry system that provide instant feedback, and using manual review of forms, the staff of the clinics and Regional Network Centers can receive feedback that reinforces training and assures the quality of the data collected and entered.
